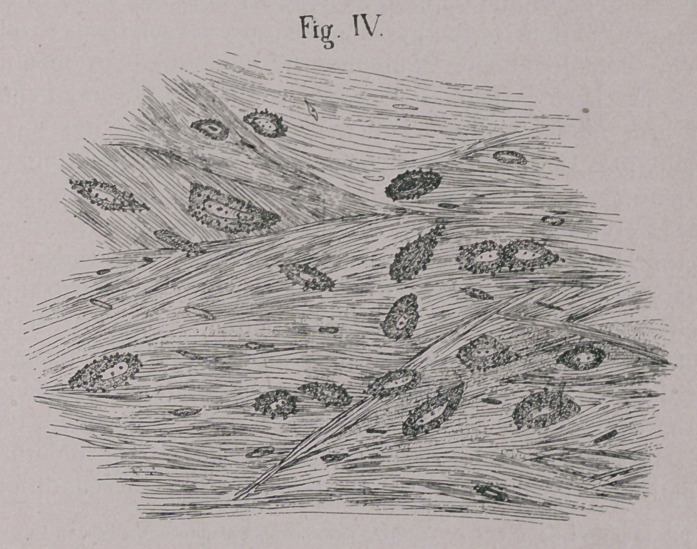# Diffuse Melanosis in a Cow

**Published:** 1893-01

**Authors:** Giuseppe Unvolette

**Affiliations:** Superintendent of the Abattoirs of Parma


### DIFFUSE MELANOSIS IN A COW.

By Dr. Giuseppe Unvolette,*
Superintendent of the Abattoirs of Parma.

On July 2d, 1890, there came to the abattoirs, from the neighborhood of Landini, a Swiss cow, brown in color, which had been used to the yoke, and had a certificate from a veterinarian that it was affected with chronic arthritis. When examined it had no fever—thermometer 38.6 degrees C.—cardiac pulsation slightly more than normal, forty-four to the minute, but with an unequal system, though not intermittent, the nutrition was good enough to send the animal for slaughter.

When the cow was slaughtered and skinned it showed in the subcutaneous connective tissue a number of small bodies from the size of a millet seed to that of a grain of wheat, black, round and more or less pedunculated. They were soft and friable and let ooze from their surface a dense liquid which stained the hands and surrounding objects black. In the muscular masses of the thighs, shoulders and back there were an innumerable quantity of these neoplasms, of variable size and form from a speck to the size of a dollar, all of a melanotic nature. It was found that all were pedunculated from the inter-muscular tissue, and that the deposit appeared as a stain, spot or tumor on the surface.

On opening the abdominal cavity it was found that the peritoneal walls were covered with a quantity of small neoplasms, analogous to those already described, from the size of a lentil to that of a pigeon's egg, all with a peduncle, and round or flattened in shape. The same lesions were found in the stomach,

*Translated.

colon, cæcum, intestines and rectum, but none were found in the epiploon or mesentery, although the mesenteric glands were infiltrated although not enlarged. The greatest deposit was in the spleen, where the tumors were as large as a man's fist, invading the splenic pulp and the centre of the organ. The deposit was softened and appeared as a colloid neoplasm. Numerous tumors were found in the uterus. The bladder, liver and kidneys were normal in size, but were studded with melanotic specks. The costal pleura and diaphragm were covered with small tumors like the peritoneum, while the lungs and mediastinum were normal. The pericardium and the heart were covered with tumors, the majority of which were found in the ventricles and on the endothelium of the pulmonary artery, while the aorta was immune.

The pigment substance from the spleen, mixed with sterilized water, was injected into two guinea pigs and a rabbit. The guinea pigs showed no symptoms of trouble except local tumefaction. In the rabbit, after a few days, there was local inflammation and suppuration, with slight fever. Six months later at the autopsy there was no trace of disease. Comparative study of the melanotic tumors of man and the horse show that they are histologically identical.

A melanotic tumor, preserved in alcohol, washed with distilled water and treated with glycerine and oil of cloves, upon examination under the microscope, showed fibrous tissue and old connective tissue cells, fusiform, oval and sometimes round, with a number of young cells with nuclei and nucleoli not yet invaded with melanotic matter. In the cells already invaded with melanotic matter, it was not possible to distinguish the external membrane from the nucleus, or the pigment mass from the protoplasm.

In an interesting case of a mare which came in the abattoir in October of the same year, the melanotic tumors invading the thoracic and abdominal cavities showed a slate gray to the eye, were solid in consistence and under the microscope showed that they were composed of fibrillar tissue, and fusiform and ovoid connective tissue cells, together with numerous embryonic cells. The fusiform cells had an elongated nucleus and nucleolus, while the ovoid, on the contrary, had two or three nuclei and brilliant nucleoli. At times both the fusiform, as well as the oval cells, were invaded by melanotic deposit, which gave their protoplasm a beautiful chocolate color, but the young cells showed but little lesion. In the majority of the cells the nucleus remained transparent and the melanotic deposit appeared as a black spot well defined. No appreciable difference could be seen in the form, volume or color of the melanotic corpuscles in the cow, mare or man.

From this study can be deducted the identity of the anatomical structure of melano-sarcoma in the cow, horse and man, which constitutes a pathological unit, and that they come from one and the same cause, which ought to be a micro-organism or figured element. The museums show neoplasms of the size of a small coin at the base of the tongue and on the heart; melanotic infusion extending to the medulla of the bones, and into the bones themselves, infringing on the spongy tissue of the femur, the vertebræ and the frontal, occipital and parietal bones. The meninges, the cerebrum, cerebellum and medulla oblongata remain normal.

The fact that the young cells are not invaded with melanotic corpuscles, or are only so partially, show that the invasion is progressive by continuity and that the sarcomatose condition is probably secondary and consecutive to the melanotic deposit.

From the various experiments made it is probable, at least admissible, that the flesh coming from animals affected with cancerous neoplasms, such as come from tuberculous animals, require the adoption of hygienic measures in the public abattoirs and exclude from consummation the flesh of animals infected with sarcoma, carcinoma, lymphadenoma, melanosis and do not permit their use when even only one organ is affected.

---

### EXPLANATION OF PLATE.

FIG. I. Melanotic corpuscles in cow. 750 diameters.

FIG. II. Melanotic corpuscles in mare.
*a.* Fusiform cells invaded with melanosis.

FIG. III. Section of melano-sarcoma in the cow. 450 diameters.
*a.* Binucleated connective tissue cells.
*b.* Embryonic cells.
*c.* Cells not yet invaded with melanosis.

FIG. IV. Section of melano-sarcoma from the skin of a man.
*a.* Cells partly invaded with melanotic corpuscles
*b.* Embroyonic cells. 450 diameters.

## Figures and Tables

**Fig. I. f1:**
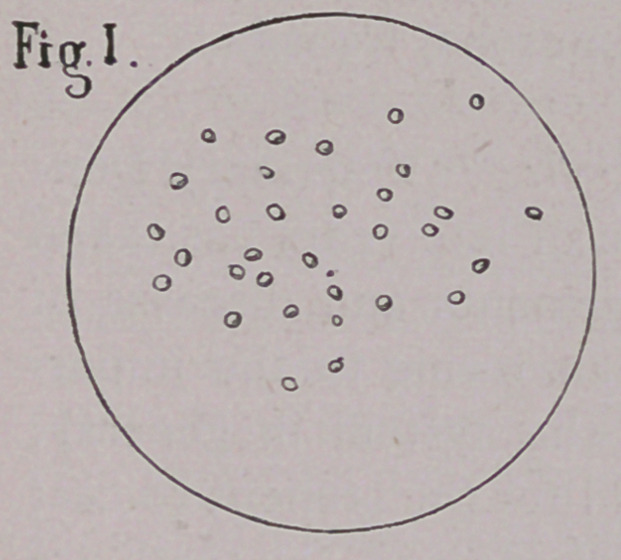


**Fig. II. f2:**
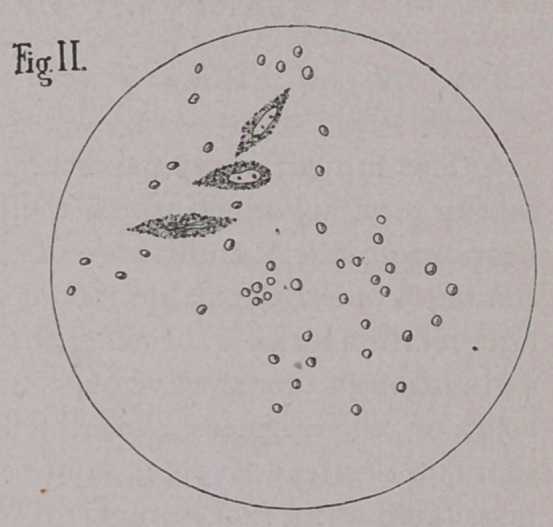


**Fig. III. f3:**
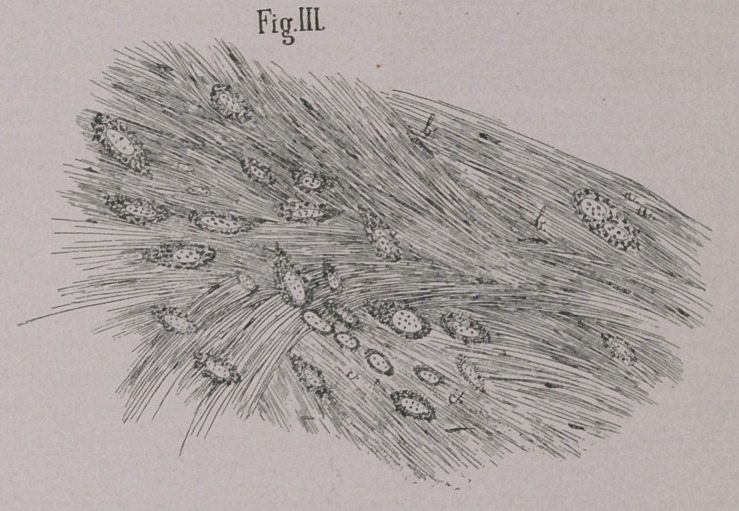


**Fig. IV. f4:**